# Stamp2 Protects From Maladaptive Structural Remodeling and Systolic Dysfunction in Post-Ischemic Hearts by Attenuating Neutrophil Activation

**DOI:** 10.3389/fimmu.2021.701721

**Published:** 2021-10-06

**Authors:** Martin Mollenhauer, Senai Bokredenghel, Simon Geißen, Anna Klinke, Tobias Morstadt, Merve Torun, Sabrina Strauch, Wibke Schumacher, Martina Maass, Jürgen Konradi, Vera B. M. Peters, Eva Berghausen, Marius Vantler, Stephan Rosenkranz, Dennis Mehrkens, Simon Braumann, Felix Nettersheim, Alexander Hof, Sakine Simsekyilmaz, Holger Winkels, Volker Rudolph, Stephan Baldus, Matti Adam, Henrik ten Freyhaus

**Affiliations:** ^1^ Department for Experimental Cardiology, Faculty of Medicine, University of Cologne, and Clinic III for Internal Medicine, University Hospital Cologne, Cologne, Germany; ^2^ Center for Molecular Medicine Cologne (CMMC), University of Cologne, Cologne, Germany; ^3^ Cologne Cardiovascular Research Center (CCRC), University of Cologne, Cologne, Germany; ^4^ Clinic for General and Interventional Cardiology/Angiology, Herz- und Diabeteszentrum Nordrhein-Westfalen, University Hospital Ruhr-Universität Bochum, Bad Oeynhausen, Germany

**Keywords:** six-transmembrane protein of prostate 2, Stamp2, Steap4, myocardial infarction, ischemia and reperfusion damage, left ventricular dysfunction, inflammation, polymorphonuclear neutrophils (PMN)

## Abstract

The six-transmembrane protein of prostate 2 (Stamp2) acts as an anti-inflammatory protein in macrophages by protecting from overt inflammatory signaling and Stamp2 deficiency accelerates atherosclerosis in mice. Herein, we describe an unexpected role of Stamp2 in polymorphonuclear neutrophils (PMN) and characterize Stamp2’s protective effects in myocardial ischemic injury. In a murine model of ischemia and reperfusion (I/R), echocardiography and histological analyses revealed a pronounced impairment of cardiac function in hearts of Stamp2-deficient- (*Stamp2^-/-^*) mice as compared to wild-type (WT) animals. This difference was driven by aggravated cardiac fibrosis, as augmented fibroblast-to-myofibroblast transdifferentiation was observed which was mediated by activation of the redox-sensitive p38 mitogen-activated protein kinase (p38 MAPK). Furthermore, we observed increased production of reactive oxygen species (ROS) in *Stamp2^-/-^* hearts after I/R, which is the likely cause for p38 MAPK activation. Although myocardial macrophage numbers were not affected by Stamp2 deficiency after I/R, augmented myocardial infiltration by polymorphonuclear neutrophils (PMN) was observed, which coincided with enhanced myeloperoxidase (MPO) plasma levels. Primary PMN isolated from *Stamp2^-/-^* animals exhibited a proinflammatory phenotype characterized by enhanced nuclear factor (NF)-κB activity and MPO secretion. To prove the critical role of PMN for the observed phenotype after I/R, antibody-mediated PMN depletion was performed in *Stamp2* ^-/-^ mice which reduced deterioration of LV function and adverse structural remodeling to WT levels. These data indicate a novel role of Stamp2 as an anti-inflammatory regulator of PMN and fibroblast-to-myofibroblast transdifferentiation in myocardial I/R injury.

## Highlights 

Herein, we investigate the role of Stamp2 in hearts subjected to ischemia and reperfusion injury. Loss of Stamp2 enhances neutrophil activation, thereby aggravating maladaptive structural remodeling and systolic dysfunction.

## Introduction

As the most severe manifestation of coronary artery disease (CAD), acute myocardial infarction (AMI) remains a leading cause of death in the western world despite an overall reduction in AMI mortality due to more aggressive approaches to coronary revascularization (1, 2). Revascularization is associated with inflammatory tissue injury at the site of ischemia and reperfusion (I/R) leading to subsequent ventricular fibrotic remodeling and heart failure (HF) (3). HF is the most common reason for hospitalization and affects around 26 million people worldwide (4).

I/R is a major trigger of polymorphonuclear neutrophils (PMN) recruitment and activation. PMN are among the first cells that infiltrate the ischemic area and accumulate in great numbers within the infarcted region. Intriguing data suggest that PMN’s level of inflammatory activation determines the extent of fibrotic scar generation and thereby recovery or persistent deterioration of cardiac function (5–7). Whereas total antibody-mediated depletion of PMN impairs infarct healing (8), targeted and more sophisticated modulation, e.g. by inhibition of neutrophil-secreted inflammatory proteins like myeloperoxidase (MPO), improves cardiac repair and function (9).

Activation of PMN stimulates fibroblast-to-myofibroblast transdifferentiation, giving rise to the major collagen-producing cell type within the heart (10). This process can be mediated by oxidative activation and phosphorylation of the p38 mitogen-activated protein kinase (p38 MAPK) which is, among others, induced by MPO and subsequent release of hypochlorous acid (HOCl) (11, 12).

The six-transmembrane protein of the prostate 2 (Stamp2), also known as six-transmembrane epithelial antigen of prostate 4 (STEAP4), has emerged as a regulator of leukocyte-driven inflammation in metabolic syndrome (13) atherosclerosis (14), and pulmonary vascular remodeling (15). Containing an NADP-oxidoreductase motif (Rossman Fold), Stamp2 is able to oxidize NADPH to NADP^+^, thereby transferring electrons through the plasma membrane to iron and copper ions (14, 16). Upon loss or dysfunction of Stamp2, NADPH accumulates within the cytosol and binds to the NADPH sensor and inhibitor of nuclear factor (NF)-κB activity NmrA-like family domain-containing protein 1 (NmRal), thereby preventing its nuclear translocation. In turn, nuclear NF-κB activity is enhanced, causing production and release of proinflammatory mediators (14).

Here, we show that Stamp2 is a novel anti-inflammatory regulator in myocardial I/R damage and omit closely related to the development of adverse ventricular remodeling and HF. *Stamp2^-/-^* mice subjected to I/R showed a pronounced inflammatory PMN activation and elevated fibroblast-to-myofibroblast transdifferentiation, revealing a role of Stamp2 as a potential target for upcoming anti-inflammatory therapies in myocardial injury.

## Methods and Materials

### Animals and Neutrophil Depletion

The generation of *Stamp2^-/-^* mice was described by Wellen et al. (13). WT and *Stamp2^-/-^* mice were used as littermates for all animal studies. Animals were kindly provided by Prof. Gökhan S. Hotamisligil (Sabri Ülker Center, Department of Molecular Metabolism and Broad Institute of Harvard-MIT and Harvard T.H. Chan School of Public Health, Boston, US). 8- to 12-week old male mice (C57/Bl6 background) were used for all animal studies. Neutrophil depletion was performed by intraperitoneal (i.p.) injection of monoclonal antibody clone 1A8 (50 µg, Stemmcell, Cologne, Germany) 1 day prior to I/R and on day 3 after I/R (8).

### Left Anterior Descending Artery Ligation

Mice were anaesthetized by intraperitoneal (i.p.) injection of 5 mg/kg bodyweight midazolam and 0.25 mg/kg bodyweight medetomidine. Analgesia was performed by i.p. injection of 0.05 mg/kg bodyweight fentanyl. Body temperature was kept constant using a rectal thermometer and an electric warming pad. To compensate evaporation, the animals received a continuous infusion of pre-warmed saline. Animals were placed in a supine position, intubated under direct laryngoscopy with a 22 gauge angiocath and ventilated using a small animal respirator (Harvard Apparatus, USA; tidal volume: 0.1 ml per 10g mouse body weight, ventilation rate: 170/min). Surgical procedures were carried out using a dissecting microscope (Leica MZ6, Leica Microsystems, Germany). After lateral thoracotomy of the fourth intercostal space, a suture (8/0 polypropylene suture, Polypro, CP Medical, USA) was placed around the left coronary artery after retraction of the left atrial appendage. The artery was ligated with a bow tie. The ligation was removed after 40 min to allow for reperfusion, the thorax was closed and the animals were allowed to recover on a warming pad.

### Echocardiographic Studies

Transthoracic echocardiography was performed using the Vevo 3100 System (VisualSonics, Toronto, Canada) (17). B-mode and M-mode recordings were performed using a MX 550S transducer (25-55 MHz) with a frame rate of 230–400 frames/s to assess LV dimensions. All images were recorded digitally and analysis was performed using the Vevo 3100-software. Ejection fraction (EF), cardiac output (CO) and global longitudinal strain were calculated as described before (18). Echocardiography was performed before surgery (baseline) and 7 days after AMI.

### Analysis of Fibrotic Area

Hearts were excised and cut along the long axis at the ligation position or, for non-infarcted animals, at the center of the left ventricle, fixed in 3.7% formaldehyde solution for 2 days and embedded in paraffin. Consecutive long axis sections of 4 μm were cut. Sections were stained with Masson Trichrome solution following standard protocols. Images were acquired using a BZ-9000 microscope (Keyence, Germany). The area of fibrosis in percent of the left ventricle was quantified using Keyence analytic software (Keyence, Germany). Mean fibrotic area of 3 sections was calculated, respectively (12).

### MPO Plasma Level

Blood was drawn into heparinized syringes in deep isoflurane anesthesia by heart puncture, followed by centrifugation for 10 min at 1,300 x g. Plasma was analyzed for MPO using a Mouse MPO ELISA (Hycult biotech, Uden, Netherlands) according to manufacturer’s instructions (12).

### DHE Staining of Ventricular Sections

Frozen heart sections were stained with dihydroethidium (DHE, 5 µM, diluted in DMSO and HBSS-buffer, ThermoFisher, Germany). The slides were incubated with DHE for 30 minutes at 37°C in the dark before pictures were taken.

### Staining for Myocardial Macrophage and PMN Infiltration

Hearts were frozen in OCT compound and cut into 6 μm sections. Frozen heart specimens were fixed with acetone. Sections were incubated with rat anti-mouse F4/80 (1:100, Abcam plc, UK) or with neutrophil Ly6G primary antibody (1:40, Hycult biotech, NL) and endogenous peroxidase activity was blocked. Secondary antibody was horseradish peroxidase (HRP)-labeled rabbit anti-rat (1:100, Dako, Glostrup, Denmark) and tertiary antibody was HRP-labeled goat anti-rabbit (1:500, Vectorlabs, Burlingame, USA) in 3% normal mouse serum, respectively. Macrophages and PMN were stained with AEC solution and tissue was counterstained with hematoxylin. Images were acquired using a BZ-9000 microscope (Keyence, Germany). Results are shown as mean number of F4/80^+^ or Ly6G^+^ cells of the LV tissue area (12).

### Immunofluorescence Staining for Myofibroblasts

Hearts were frozen in OCT compound and cut to 6 μm longitudinal sections. Sections were thawed, fixed with 3.7% formaldehyde solution and were blocked with 10% mouse serum. Slides were treated with 0.1% Triton X-100 and incubated with primary antibody against α–smooth muscle actin (α-SMA; 1:200, rabbit IgG, ab5694, Abcam, Cambridge, UK) and discoidin domain-containing receptor 2 (DDR-2; 1:50, goat IgG, sc7555, Santa Cruz, Texas, USA) respectively for 1 hr at room temperature in PBS with 0.1% Triton-X100 and 10% mouse serum. Secondary antibody was Alexa Fluor-594 chicken-anti-rabbit IgG and Alexa Fluor-488 chicken-anti-goat IgG (Invitrogen) and nuclei were stained with DAPI. Confocal imaging was performed using a TCS SP8 confocal microscope (Leica Microsystems, Germany).

### Fibroblast Isolation and Characterization

Mice subjected to I/R were sacrificed, ventricles were removed and washed in sterile HEPES-buffered Tyrode’s solution (135 mM NaCl, 4 mM KCl, 0.3 mM NaH2PO4, 1 mM MgCl2, 10 mM HEPES, 2 g/l glucose, pH = 7.3, Sigma-Aldrich, St. Louis, USA). Ventricles were minced and digested in 0.1 g/l Liberase/Tyrode solution (Liberase TM research grade, Roche, Basel, Switzerland) for 10 minutes at 37°C. The supernatant was collected and the digestion step repeated 6 times. The supernatant was filtered (40 μm cell strainer, Thermo Fisher Scientific, Waltham, USA), centrifuged and the fibroblasts were resuspended in DMEM supplemented with 10% FCS. The cells were again centrifuged and further processed for immunoblotting.

For mRNA investigations, hearts were isolated from 10-12 week old WT and *Stamp2^-/-^* mice. Ventricles were cut into 1-2 mm^2^ pieces and digested in a semi-automated dissociation process following manufacturer’s protocol (GentleMACS Dissociator and Multi Tissue Dissociation Kit 2, Miltenyi Biotec, Bergisch-Gladbach, Germany). Cell suspension was resuspended in DMEM+ Glutamax (PAN-Biotech, Aidenbach, Germany) supported by 10% fetal calf serum (FCS), 1% Penicillin/Streptomycin and 0,1% Fibroblast Growth Factor (Recombinant Human FGF-basic (154 a.a.) Peprotech, Rocky Hill, NJ, USA). Cells were transferred to 6-well plates pre-coated with 1% gelatin at 37°C and 5% CO_2_. Cells were split at 70-80% confluency. In 3^rd^ passage, cells were harvested by adding 800µl of RNA Lysis Buffer T to each well and Stamp2 mRNA expression analyses were performed.

### PMN Isolation and Characterization

PMN isolation was performed following a modified standard protocol by English and Andersen (19). In short, murine EDTA blood was taken by cardiac puncture. Blood was applied on a double layer of Histopaque 1119/1077 (Sigma Aldrich, Germany) and centrifuged at 700g for 30 min without break. Granulocytes located between Histopaque 1119 and Histopaque 1077 were carefully isolated and washed two times with HBSS. Cell number was counted. For Stamp2 expression analyses 100,000 cells were used. For NF-κB activity analyses cells were lysed in RIPA buffer. For MPO secretion analyses 100,000 cells were treated with PMA (Sigma-Aldrich, Germany) 100ng/ml or with saline for 2 hours at 37°C in HBSS. Supernatants were collected and vaporized by SpeedVac Vacuum centrifugation for 24 hours. Residues were dissolved in 100µl H_2_O and MPO levels were assessed (12).

### Immunoblotting

Protein extraction and immunoblotting were performed according to standard protocols (20). Briefly, membranes were incubated with the following antibodies overnight at 4°C: anti-GAPDH (1:1000; Santa Cruz), anti-p65 (1:5000; Abcam ab7970), Phospho-NF-κB p65 (Ser536) (1:1000 CellSignaling), p38 MAP kinase (CellSignaling, 1:100, New England Biolabs, Frankfurt, Germany), p-p38 MAP kinase (CellSignaling, 1:100, New England Biolabs). After incubation with appropriate secondary antibodies for 1 hour, chemiluminescence was detected using a Fusion FX (Vilber Lourmat) imaging system and quantified with Quantity One (BioRad) (20).

### mRNA Expression Analyses

RNA-Isolation was performed by using the peqGOLD Total RNA Kit (VWR, Darmstadt, Germany). mRNA was converted to cDNA. Stamp2 mRNA expression was investigated by quantitative real-time PCR using the following primers: Stamp2 (NM_054098.3) -fwd: TCAAATGCGGAATACCTTGCT, -rev: GCATCTAGTGTTCCTGACTGGA; 18S ribosomal RNA (NR_003278.3) -fwd: GTAACCCGTTGAACCCCATT, -rev: CCATCCAATCGGTAGTAGCG.

### White Blood Cell Count

EDTA blood was taken by cardiac puncture from untreated WT and *Stamp2^-/-^* male mice and immediately analyzed by the 5-Part hematology-system Sysmex XN (Sysmex, Gemany).

### Infarct Size Determination

Hearts were excised and the aortic arch was cannulated to perform perfusion with PBS supplemented with 50I.U. heparin/ml in a retrograde manner. Consequently, the knot that had been used for initial ligation was closed and perfusion with Evan’s blue dye was performed to stain healthy tissue. Afterwards, hearts were cut into 1mm slides and incubated in 2,3,5-triphenyl-tetrazolium chloride solution. Healthy tissue was identified as blue area, area at risk as red area and infarcted tissue as white area using the BZ2-Analyser software (Keyence).

### Heart Digestion and Flow Cytometry

For flow cytometry analysis, hearts were perfused *via* the left ventricular cavity with 10ml of PBS supplemented with heparin (50I.E./ml), immediately mechanically disrupted in digestion buffer (450u/ml collagenase I (Sigma Aldrich, St. Loius, MO, USA), 125u/ml collagenase XI (Sigma Aldrich), 60u/ml hyaluronidase (Sigma Aldrich) and 60u/ml DNAse I (Thermo Fisher Scientific, Waltham, MA, USA) in HBSS) and digested for 1 hour at 30°C. The single cell suspension was stained with a cocktail of antibodies including CD45, CD11b, CD64, CCR2 and TIMD4 (an antibody list including clones, fluorescent dyes and manufacturer data can be found in the supplementary material). Fluorescence minus one controls (FMO) for each marker were used to assure correct compensation. The Zombie UV fixable kit (Biolegend, San Diego, CA, USA) was used to assure cell viability. Stained cells were analyzed with a Cytek Aurora flow cytometer (Cytek, Fremont, CA, USA). Cells were gated for single, viable leukocytes. After exclusion of lymphocytes and monocytes, macrophages were identified as CD64^+^ CD11b^+^ cells. Timd4 was used as a marker for long-term residual macrophages, CCR2 as marker for monocyte derived macrophages (21).

### Statistical Analysis

In all cases, investigators were blinded for the genotype or treatment group of the respective animals or samples. Results are expressed as mean ± SEM. Normal distribution was tested for by either Kolmogorov-Smirnov- or Shapiro-Wilk-Test. For parametric data, comparative analysis was performed using Unpaired Student’s t-test or Two-way ANOVA followed by Tukey *post-hoc* test. For nonparametric data, either Mann-Whitney- or Kruskal-Wallis-test followed by Dunn’s *post-hoc* test was performed. A *P-*value of *< 0.05* was considered statistically significant. Graphs were created with GraphPad Prism 7.0a. All statistical calculations were carried out using GraphPad Prism 7.0a (GraphPad). *P < 0.05, **P < 0.01, ***P < 0.001.

## Results

### Stamp2 Deficiency Further Decreases Systolic Left Ventricular Function in a Murine Model of I/R

Given the abundance of Stamp2 in leukocytes and its potential anti-inflammatory role in atherosclerosis, we investigated the effects of Stamp2 deficiency on left ventricular (LV) function in a murine model of myocardial I/R injury. Thus, *Stamp2^-/-^* and WT mice were subjected to ligation of the left anterior descending artery (LAD) for 40 minutes followed by myocardial reperfusion for up to 7 days.

**Figure 1:**
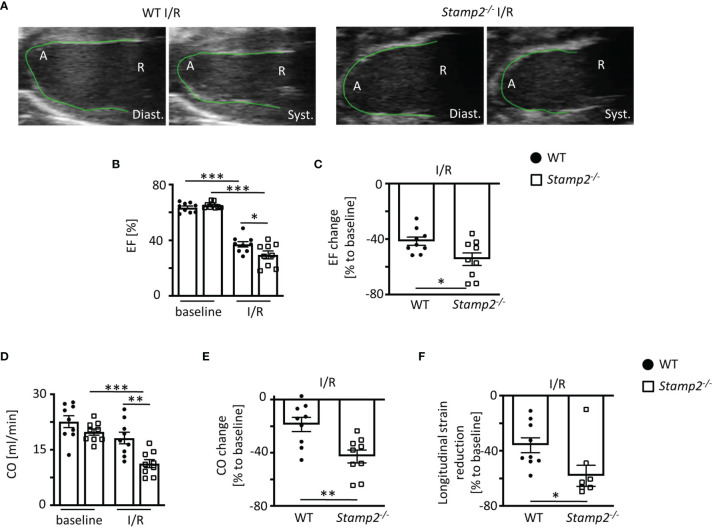
Left ventricular (LV) function is impaired in *Stamp2* ^-/-^ mice when subjected to ischemia and reperfusion injury (I/R). **(A)** Representative echocardiographic recordings indicating enhanced reduction of LV function in *Stamp2^-/-^* hearts as compared to WT after 7 days of I/R (A=Apex, R= Aortic root; Diast.=diastole Syst.=systole). **(B)** Total ejection fraction (EF, n=9/9/9/9) and **(C)** change of EF in % to baseline (n=9/9). **(D)** Total cardiac output (CO, n=9/9/9/9) and **(E)** change of CO in % to baseline (n=9/9) as assessed by echocardiography. **(F)** Global longitudinal strain reduction after subjection to I/R (n=9/9). Baseline echocardiography was directly performed before MI induction and I/R echocardiography at day 7. Graphs show Mean ± SEM. Significance was determined by two-way ANOVA followed by Tukey *post-hoc* test **(B, D)** or by unpaired Student’s t-test **(C, E, F)**. *P < 0.05, **P < 0.01, ***P < 0.001.

In *Stamp2^-/-^* mice, echocardiographic analyses (representative recordings are shown in **Figure 1A**, full echocardiographic recordings are provided as **Supplemental Videos 1** and **2** in the supplemental material) revealed a substantially stronger reduction of LV EF as compared to WT (mean reduction in **Figure 1B** and reduction within the same animal in **Figure 1C**) Accordingly, total CO (**Figure 1D**, change of CO in **Figure 1E**) was more significantly reduced in *Stamp2^-/-^* hearts. In line with these observations, global longitudinal strain reduction was more pronounced in *Stamp2^-/-^* hearts after I/R as compared to WT (**Figure 1F**). Moreover, thinning of the left ventricular wall was significantly more pronounced in *Stamp2^-/-^* animals (**Supplemental Figures S1A, B**). Of note, baseline characterization of heart function and vital parameters under isoflurane narcosis revealed a reduced heart rate in *Stamp2^-/-^* animals (**Supplemental Figure S2**). Altogether, this data indicates a prominent role of Stamp2 in LV function after ischemic injury.

### Stamp2 Deficiency Promotes Left Ventricular Fibrotic Remodeling

**Figure 2:**
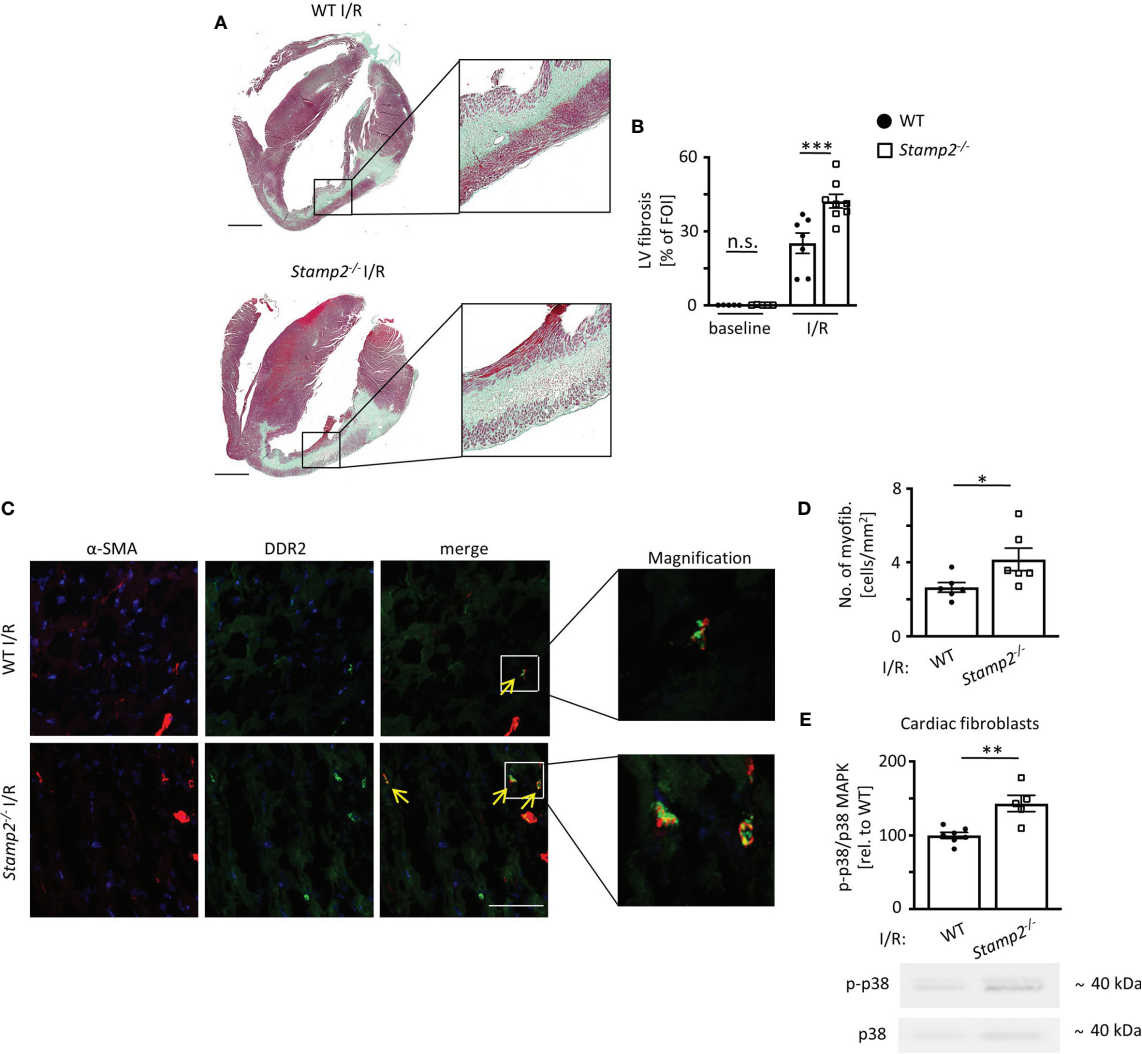
Stamp2 deficiency aggravates I/R-induced LV fibrotic remodeling. **(A)** Representative images of Masson’s trichrome-stained cardiac sections of WT and Stamp2^-/-^animals 7 days after I/R. Scale bar=1mm. **(B)** Quantification of left ventricular fibrotic areas stained in green (n=5/5/7/8). **(C)** Representative immunofluorescence stainings imaged by confocal microscopy for the myofibroblast marker α-SMA (α-smooth muscle actin; red), for the fibroblast marker DDR-2(discoidin domain-containing receptor 2; green) and for nuclei (DAPI, blue). Scale bar=50μm. **(D)** Quantitative analaysis of myofibroblasts within the peri-infarct region (n=6/6). **(E)** Relative phosphorylation of p38 MAPK (p-p38/p38 MAPK) in isolated primary fibroblasts from hearts 3 days post I/R (n=7/5). Graphs show Mean ± SEM. Full blots are shown in **Supplemental Figure S4**. Significance was determined by two-way ANOVA followed by Tukey *post-hoc* test **(B)** or by unpaired Student’s t-test **(D, E)**. *P < 0.05, **P < 0.01, ***P < 0.001. n.s., not significant.

Post-infarct LV remodeling includes fibrotic scar formation that promotes development of HF (22). To assess the impact of Stamp2 on fibrotic remodeling, Masson’s trichrome staining was performed, which revealed significantly larger areas of collagen deposition in cardiac sections of *Stamp2^-/-^* mice as compared to WT (**Figures 2A, B** *)*.

As myofibroblasts are the major source of collagen in the infarcted myocardium (23), myofibroblast accumulation was analyzed by immunoreactivity to the fibroblast marker DDR-2 and to the myofibroblast marker α-SMA 7 days post I/R. These experiments revealed that the number of myofibroblasts was significantly elevated in the peri-infarct region of *Stamp2^-/-^* hearts as compared to WT (**Figures 2C, D** *)*. Fibroblast-to-myofibroblast transdifferentiation in the context of inflammation is driven by activation and phosphorylation of the p38 MAPK pathway (12, 24). To test whether this process is involved in the observed phenotype, we isolated primary cardiac fibroblasts from *Stamp2^-/-^* and WT hearts. Immunoblottings of isolated primary cardiac fibroblasts after I/R demonstrated increased p38 MAPK phosphorylation in *Stamp2^-/-^* cells as compared to WT controls (**Figure 2E** *, full unedited gel shown in* **Supplemental Figure S8**). Of note, total infarct size was unchanged in *Stamp2^-/-^* hearts (**Supplemental Figure S3**).

### Stamp2 Deficiency Promotes Myocardial PMN Infiltration Upon I/R

**Figure 3:**
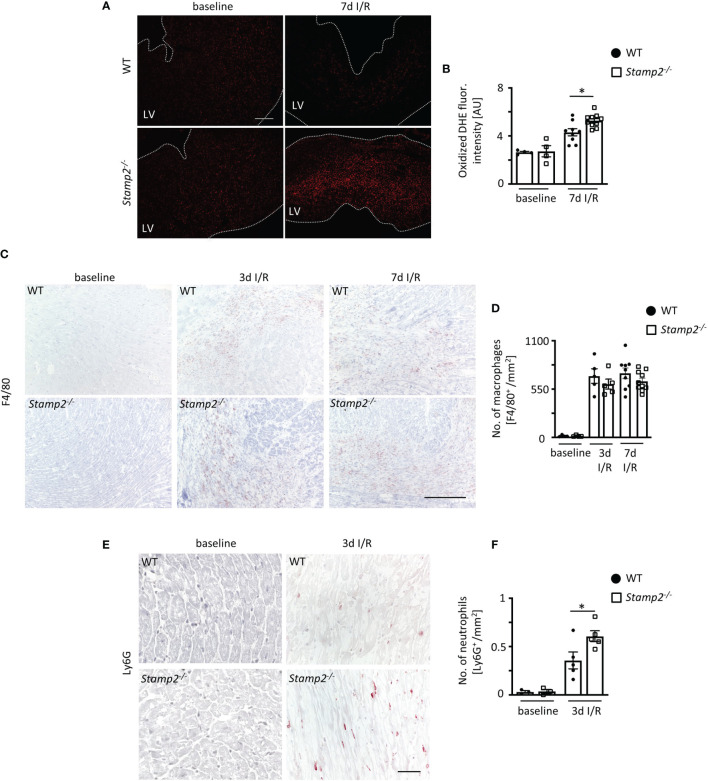
Stamp2 regulates myocardial PMN infiltration after I/R. **(A)** Representative stainings of oxidized dihydroethidium (DHE) and **(B)** quantitative analysis of ROS production in myocardial sections 7 days after I/R induction (n=4/4/7/8). Scale bar=200 μm. **(C)** Representative left ventricular immunohistological stainings for the pan-macrophage marker F4/80 (brown) and **(D)** quantitative analysis of macrophage numbers within the infarct region (n=4/4/5/5/9/10). Scale bar= 200 μm. **(E)** Representative left ventricular immunohistological stainings for the PMN marker Ly6G (brown) and **(F)** quantitative analysis of neutrophil numbers within the infarct region 3 days after I/R induction (n=3/3/5/5). Scale bar = 50 μm. Graphs show Mean ± SEM. Significance was determined by two-way ANOVA followed by Tukey *post-hoc* test. *P < 0.05.

Leukocyte activation upon myocardial I/R injury is associated with cardiac fibrotic remodeling and is the main contributor to profibrotic fibroblast-to-myofibroblast transdifferentiation (24). Apart from cytokines and growth factors, p38 MAPK signaling can be driven by PMN-derived reactive oxygen species (ROS) (12, 25). Thus, we analyzed the oxidation of dihydroethidium (DHE) in cardiac sections as an indicator for superoxide release. Fluorescence intensity of oxidized DHE was significantly elevated in *Stamp2^-/-^ vs.* WT hearts after I/R, demonstrating increased ventricular ROS production (**Figures 3A, B** *)*.

Given the anti-inflammatory role of Stamp2 in macrophages, we expected an increased abundance of these cells within the infarct region of *Stamp2^-/-^* mice. However, despite increased macrophage numbers upon I/R in both genotypes, numbers were not different in *Stamp2^-/-^* mice (**Figures 3C, D** *)*. This could be further confirmed by flow cytometric analyses indicating equal numbers and proportions of macrophage populations in both genotypes 3 days after I/R induction (**Supplemental Figure S4**).

PMN are among the first ROS-producing cells invading the injured myocardium. Their inflammatory activation has been closely linked to ventricular remodeling (5). Consequently, we quantified LV PMN abundance by immunohistochemical stainings for the PMN marker Ly6G. Strikingly, PMN numbers were substantially increased in *Stamp2^-/-^* hearts *vs.* WT 3 days after I/R (**Figures 3E, F** *)*.

### Stamp2 Regulates Proinflammatory PMN Activation and Myeloperoxidase Secretion

**Figure 4:**
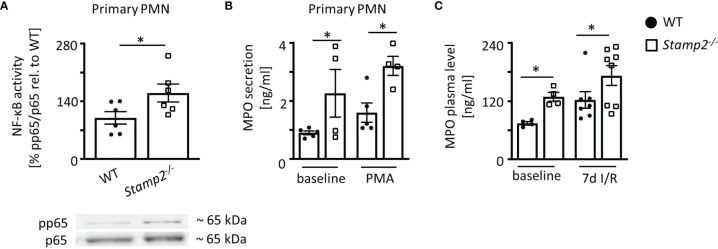
Proinflammmatory activation of PMN by Stamp2 deficiency. **(A)** Immmunoblotting of p65-phosphorylation (pp65) *vs.* total p65 expresssion as an indicator of NF-κB activity (n=6/6). **(B)** Myeloperoxidase (MPO) secretion into the supernatant of isolated PMN as assessed by ELISA with and without inflammatory stimulation (PMA 100ng/ml; n=5/4/5/4). **(C)** MPO plasma levels in mice at baseline and subjected to 7 days of I/R as assessed by ELISA (n=4/4/7/8). Graphs show Mean ± SEM. Full blots are shown in **Supplemental Figure S5**. Significance was determined by two-way ANOVA followed by Tukey *post-hoc* test **(B, C)** or by unpaired Student’s t-test **(A)**. *P < 0.05.

Apart from controlling myocardial PMN infiltration in the context of I/R, Stamp2 may also directly alter cellular responses. Given that Stamp2 is abundantly expressed in murine PMN (**Supplemental Figures S5, S6** *)*, Stamp2-mediated PMN activation might be closely associated with myocardial infarction, subsequent post-infarct remodeling and scar formation (8, 26). To characterize cellular responses to Stamp2 deficiency, we isolated primary PMN from *Stamp2^-/-^* and WT mice. Stamp2 deficiency led to pronounced NF-κB activity in isolated PMN as demonstrated by enhanced phosphorylation of its subunit RelA (p65) (**Figure 4A** *)*, which is closely associated with enhanced immune responses, leukocyte activation and cytokine secretion (27). Enhanced proinflammatory activation could be further confirmed *ex vivo* showing that Stamp2 deficiency elevated MPO secretion from primary isolated PMN, which reflects pro-inflammatory granule release (28) (**Figure 4B** *, full unedited gel shown in* **Supplemental Figure S9**).

Plasma levels of PMN-derived MPO were elevated in *Stamp2^-/-^* animals both at baseline conditions and after subjection to I/R (**Figure 4C**). To rule out pre-existing leukocytosis in *Stamp2^-/-^* animals, blood counts were analyzed. These demonstrated equal leukocyte numbers (white blood cell count, WBC, **Supplemental Figure S7**) in both genotypes indicating that enhanced myocardial PMN infiltration and systemic MPO levels after I/R were due to enhanced PMN activation.

### PMN Depletion Abolishes the Effect of Stamp2 Deficiency on I/R-Mediated Fibrotic Remodeling and LV Function

**Figure 5:**
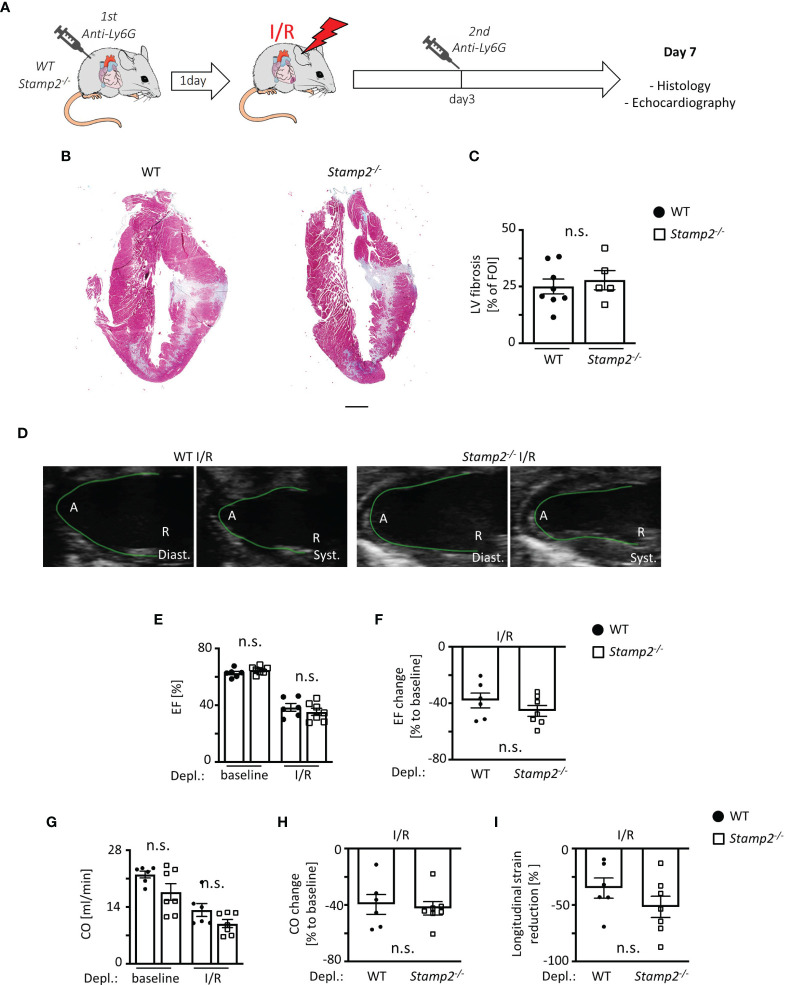
PMN depletion rescues impaired LV function in *Stamp2^-/-^* mice after I/R. **(A)** Schematic overview of PMN depletion by i.p. injection of an anti-Ly6G antibody 1 day prior to- and 3 days after subjection to I/R (13). **(B)** Representative images of Masson’s trichrome-stained cardiac sections of WT- and *Stamp2^-/-^* animals 7 days after I/R with **(C)** assessment of the LV fibrotic areas stained in green/grey (n=8/5). Scale bar=1mm **(D)** Representative echocardiographic recordings of WT- and *Stamp2^-/-^* hearts (A=Apex, R=Aortic root; Diast.=diastole, Syst.=systole). **(E)** Total ejection fraction (EF, n=6/7/6/7) and **(F)** change of EF in % to baseline (n=6/7). **(G)** Total cardiac output (CO, n=6/7/6/7) and **(H)** change of CO in % to baseline as assessed by echocardiography (n=6/7). **(I)** Longitudinal strain reduction after subjection to I/R (n=6/7). Baseline echocardiography was directly performed before MI induction and I/R echocardiography at day 7. Graphs show Mean ± SEM. Significance was determined by two-way ANOVA followed by Tukey *post-hoc* test **(E, G)** or by unpaired Student’s t-test **(C, F, H, I)**. n.s., not significant.

To investigate whether the impairment of LV function by Stamp2 deficiency is causally linked to enhanced PMN activation and infiltration, WT- and *Stamp2^-/-^* mice were subjected to Ly6G antibody-mediated PMN depletion (injection schematic is shown in **Figure 5A**) (8). Intriguingly, PMN depletion completely reversed the maladaptive phenotype of Stamp2 deficiency after I/R. This included attenuated LV fibrotic remodeling (**Figures 5B, C** *)* and LV function. In detail, the *Stamp2^-/–^* mediated alterations in total EF (**Figure 5E**; representative recordings are shown in **Figure 5D**, full echocardiographic recordings are provided as **Supplemental Videos 3** and **4** in the supplemental material), mean EF reduction (**Figure 5F**) as well as total CO (**Figure 5G**), mean CO reduction (**Figure 5H**) and global longitudinal strain reduction (**Figure 5I**) were blunted upon PMN depletion.

**Figure 6:**
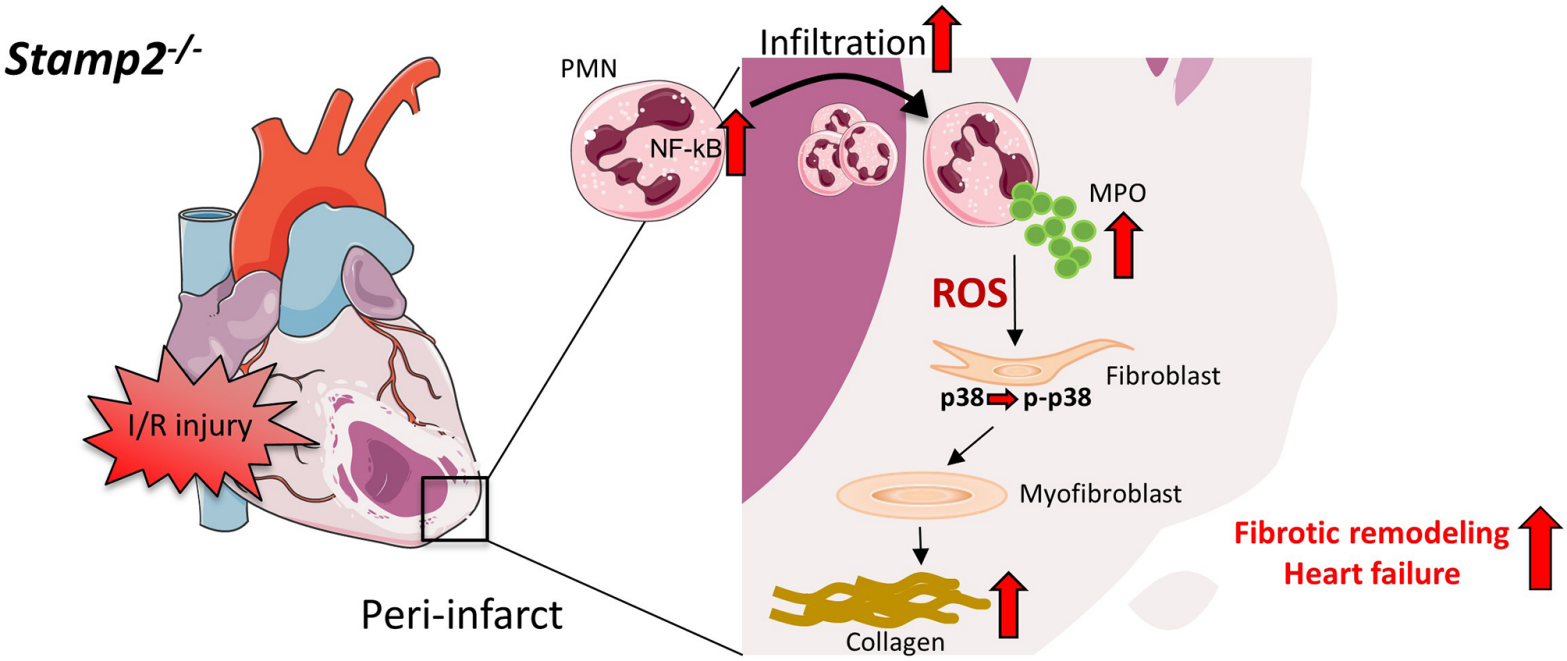
Schematic overview of PMN activation and fibrotic remodeling in *Stamp2^-/-^* mice after I/R. Stamp2 deficiency results in NmRal-mediated proinflammatory NF-κB activation (11, 15) which subsequently induces enchanced myocardial PMN infiltration after I/R injury. PMN degranulation and MPO secretion are elevated in *Stamp2^-/-^* PMN, thereby activating the p38-MAPK pathway and inducing fibroblast-to-myofibroblast transdifferentiation. In turn, enchanced collagen deposition promotes maladaptive structural remodeling finally leading to loss of LV function. PMN: polymorphonuclear neutrophils, Stamp2: six transmembrane protein of prostate 2, NF-κB: nuclear factor ‘kappa-light-chain-enchancer’ of activated B-cells, NmRal: NmrA-like family domain- containing protein 1, MPO: myeloperoxidase, p-p38/p38: phosphor*/* p38 MAP kinase. Figures were produced using Servier Medical Art (http://www.servier.com/).

Taken together, we herein demonstrate that Stamp2 deficiency leads to enhanced inflammatory PMN activation upon I/R injury resulting in pronounced fibroblast-to-myofibroblast transdifferentiation. These cellular alterations promote maladaptive fibrotic remodeling, ultimately resulting in the loss of LV function (**Figure 6**). These data put Stamp2 in a central position controlling PMN functions to protect from adverse LV remodeling after I/R injury.

## Discussion

Herein, we show that Stamp2 deficiency promotes adverse LV structural remodeling after myocardial I/R injury by elevating proinflammatory PMN activation. Studies of a murine model of myocardial I/R injury reveal that loss of Stamp2 enhances (i) inflammatory activation of PMN in mice subjected to I/R damage resulting in (ii) enhanced ventricular fibrosis by transdifferentiation of fibroblasts to myofibroblasts ultimately causing (iii) reduced LV function.

The inflammatory activation of leukocytes, in particular neutrophils, has long been regarded as a crucial mechanistic component to myocardial I/R damage (29, 30). Although the underlying mechanisms are diverse and still not completely understood (31), clinical trials targeting pro-inflammatory pathways (COLCOT, LoDoCo, CANTOS) (32–34), emphasize the clinical need and the feasibility to target and modulate innate immune responses to prevent myocardial damage.

Stamp2 has emerged as an anti-inflammatory regulator of the innate immunity by suppressing inflammatory cytokine expression (13). Furthermore, endothelial expression of leukocyte-recruiting cell surface proteins like ICAM-1 und VCAM-1 is enhanced in Stamp2 deficiency (35), a mechanism which has been closely linked to myocardial infarct healing und -function (25). In particular, the role of macrophage activation in the context of Stamp2 deficiency was reported in various pathologies like atherosclerosis (14), pulmonary hypertension (15), adipose tissue insulin resistance (36) and prostate cancer (37). Hitherto, a role of Stamp2 in PMN activation in cardiovascular disease was unknown. We show for the first time, that Stamp2 is a regulator of PMN infiltration and myocardial healing after I/R injury by modulating NF-κB activation. So far, this mechanism has been described only in atherosclerotic macrophages due to Stamp2´s NADPH-oxidizing properties (14, 37) although NF-κB regulation differs between PMN and mononuclear cells (38).

Stamp2 deficiency induces PMN degranulation and secretion of MPO, a pro-inflammatory heme enzyme which is the most abundant protein in granules of PMN (28) and responsible for maladaptive ventricular remodeling after I/R injury (12). The molecular mechanisms of Stamp2 in regulating PMN degranulation remain elusive although it is tempting to speculate that, given the importance of NADPH-oxidases in PMN priming and degranulation (39), Stamp2-mediated oxidation of NADPH might be the driving factor (14). Of note, enhanced MPO secretion could be detected not only in unstimulated isolated PMN but also in untreated *Stamp2^-/-^* animals, indicating enhanced PMN activation even under baseline conditions.

Ventricular fibroblast activation and fibrotic remodeling are significantly pronounced in *Stamp2^-/-^* animals finally resulting in severe impairment of LV function. Myofibroblasts are the main cellular contributors to ventricular fibrosis (22). In *Stamp2^-/-^* animals, MPO plasma levels are significantly higher upon myocardial injury. We have shown previously that the major MPO-derived species hypochlorous acid (HOCl) (40) leads to activation of p38 MAPK that in turn promotes fibroblast transdifferentiation and results in enhanced ventricular fibrotic remodeling (11). The importance of these fibrotic mechanisms for heart function is further underlined by the unaltered infarct size in *Stamp2^-/-^* animals upon I/R injury.

As this study was performed in a small animal model, a number of limitations may apply when translating the present findings to human pathology. Given the mechanistic complexity of involved cell types, inflammatory stimuli, growth factors, cell death- and hypertrophic signaling pathways in myocardial post-infarct healing, it has to be considered that PMN may not be the exclusive cellular mediators by which Stamp2 deficiency promotes fibrotic remodeling. Macrophages are important for structural remodeling post infarction (41) and it cannot be ruled out that Stamp2 influences macrophage activation in this regard (14). However, RNA-seq data revealed low Stamp2 expression in macrophages as compared to PMN at baseline levels and after myocardial infarction (7). Furthermore, infiltrating macrophage numbers and populations did not differ between WT- and *Stamp2^-/-^* hearts after I/R injury indicating a minor role of Stamp2 in macrophages in infarct healing.

Stamp2-mediated endogenous effects on fibroblasts could not be fully disclosed since Stamp2 mRNA is expressed in activated cardiac myofibroblasts. Nonetheless, in comparison, isolated cardiac fibroblasts showed lower Stamp2 expression than primary PMN. Accordingly, specific PMN depletion by Ly6G antibody injection, a well-established method for studying PMN in myocardial pathologies (8), completely reversed *Stamp2^-/-^* mediated functional LV impairment and enhanced fibrosis, underlining the prominent role of PMN for the observed phenotype in Stamp2 deficiency.

The reduction in heart rate in *Stamp2^-/-^* animals compared to WT animals at baseline conditions might furthermore suggest an unknown effect of Stamp 2 on the cardiac conduction system and demands further investigation.

Stamp2 expression has been inversely correlated with the pathological severity of atherosclerosis (14), pulmonary hypertension (15) and obesity (36). In view of a clinical translation, the assessment of Stamp2 expression in PMN might therefore emerge as a marker for clinical outcome in patients after myocardial infarction. Furthermore, anti-inflammatory effects of pharmacological Stamp2 activation or augmented expression may be beneficial as novel therapeutic strategies. Such an effect has been recently reported for the AMP-activated protein kinase (AMPK) activator cilostazol in nonalcoholic fatty liver disease (42). Moreover, in a setting of acute myocardial injury for 2 hours, treatment with the biguanide metformin reduced cardiomyocyte apoptosis in a Stamp2-dependent manner (43). The development of more specific compounds for pharmacological Stamp2 regulation in cardiovascular diseases is therefore an interesting goal for future studies.

In conclusion, the current data reveal that absence of Stamp2 adversely affects myocardial function after I/R injury. Mechanistically, deficiency of Stamp2 induces pro-inflammatory activation and degranulation of PMN which subsequently leads to enhanced LV fibrotic remodeling *via* activation of fibroblast-to-myofibroblast transdifferentiation (**Figure 6**). These results not only indicate that Stamp2 is a novel regulator of the inflammatory response in ischemic cardiomyopathy but also point to Stamp2 activation as a potential pharmacological target.

## Data Availability Statement 

The original contributions presented in the study are included in the article/**Supplementary Material**. Further inquiries can be directed to the corresponding authors.

## Ethics Statement

All animal studies were approved by the local Animal Care and Use Committees (Ministry for Environment, Agriculture, Conservation and Consumer Protection of the State of North Rhine-Westphalia: State Agency for Nature, Environment and Consumer Protection (LANUV), NRW, Germany) and follow ARRIVE (Animal Research: Reporting of In Vivo Experiments) guidelines.

## Author Contributions

The author contributions are as follows: MMo, SeB, MA, and HF designed the project, performed experiments and statistical analysis and prepared the manuscript. AK, TM, SSt, WS, EB, MV, SR, DM, SiB, SG, FN, AH, SSi and HW performed experiments and provided suggestions on the project. VR and StB provided substantial suggestions on the project and critically revised the manuscript. SeB and HF supervised the project. MT, MMa, JK and VP substantially performed revision experiments. SG substantially performed revision experiments and wrote the revised manuscript. MM supervised the project and wrote the manuscript. All authors contributed to the article and approved the submitted version.

## Funding

This work was funded by the Deutsche Forschungsgemeinschaft DFG (RU 16783-3 and 360043781 - GRK 2407 to VR, 360043781 - GRK 2407 to SB, MO 3438/2-1 to MM and Grant. No. 397484323 - TRR259 to SB, HW and MA) and the Center for Molecular Medicine Cologne funding (Baldus B-02). HF was funded by the Köln Fortune Program of the University of Cologne (254/2014, 240/2017), and by the German Foundation of Heart Research (F/45/15, F37/17).

## Conflict of Interest

The authors declare that the research was conducted in the absence of any commercial or financial relationships that could be construed as a potential conflict of interest.

## Publisher’s Note

All claims expressed in this article are solely those of the authors and do not necessarily represent those of their affiliated organizations, or those of the publisher, the editors and the reviewers. Any product that may be evaluated in this article, or claim that may be made by its manufacturer, is not guaranteed or endorsed by the publisher.

## References

[1] McManus DD Gore J Yarzebski J Spencer F Lessard D Goldberg RJ. Recent Trends in the Incidence, Treatment, and Outcomes of Patients With STEMI and NSTEMI. Am J Med (2011) 124:40–7. doi: 10.1016/j.amjmed.2010.07.023 PMC3011975 21187184

[2] Reed GW Rossi JE Cannon CP. Acute Myocardial Infarction. Lancet (London England) (2017) 389:197–210. doi: 10.1016/S0140-6736(16)30677-8 27502078

[3] Suthahar N Meijers WC Silljé HHW de Boer RA. From Inflammation to Fibrosis—Molecular and Cellular Mechanisms of Myocardial Tissue Remodelling and Perspectives on Differential Treatment Opportunities. Curr Heart Fail Rep (2017) 14:235–50. doi: 10.1007/s11897-017-0343-y PMC5527069 28707261

[4] Ponikowski P Anker SD AlHabib KF Cowie MR Force TL Hu S. Heart Failure: Preventing Disease and Death Worldwide. ESC Hear Fail (2014) 1:4–25. doi: 10.1002/ehf2.12005 28834669

[5] Puhl S-L Steffens S. Neutrophils in Post-Myocardial Infarction Inflammation: Damage *vs*. Resolution? Front Cardiovasc Med (2019) 6:25. doi: 10.3389/fcvm.2019.00025 30937305 PMC6431642

[6] Swirski FK Nahrendorf M. Leukocyte Behavior in Atherosclerosis, Myocardial Infarction, and Heart Failure. Science (2013) 339:161–6. doi: 10.1126/science.1230719 PMC3891792 23307733

[7] Vafadarnejad E Rizzo G Krampert L Arampatzi P Arias-Loza A-P Nazzal Y. Dynamics of Cardiac Neutrophil Diversity in Murine Myocardial Infarction. Circ Res (2020) 127:e232–e249. doi: 10.1161/CIRCRESAHA.120.317200 32811295

[8] Horckmans M Ring L Duchene J Santovito D Schloss MJ Drechsler M. Neutrophils Orchestrate Post-Myocardial Infarction Healing by Polarizing Macrophages Towards a Reparative Phenotype. Eur Heart J (2016) 241:ehw002. doi: 10.1093/eurheartj/ehw002 28158426

[9] Ali M Pulli B Courties G Tricot B Sebas M Iwamoto Y. Myeloperoxidase Inhibition Improves Ventricular Function and Remodeling After Experimental Myocardial Infarction. JACC Basic to Transl Sci (2016) 1:633–43. doi: 10.1016/j.jacbts.2016.09.004 PMC6113523 30167547

[10] Humeres C Frangogiannis NG. Fibroblasts in the Infarcted, Remodeling, and Failing Heart. JACC Basic to Transl Sci (2019) 4:449–67. doi: 10.1016/j.jacbts.2019.02.006 PMC6610002 31312768

[11] Midwinter RG Vissers MC Winterbourn CC. Hypochlorous Acid Stimulation of the Mitogen-Activated Protein Kinase Pathway Enhances Cell Survival. Arch Biochem Biophys (2001) 394:13–20. doi: 10.1006/abbi.2001.2530 11566022

[12] Mollenhauer M Friedrichs K Lange M Gesenberg J Remane L Kerkenpaß C. Myeloperoxidase Mediates Postischemic Arrhythmogenic Ventricular Remodeling. Circ Res (2017) 121(1):56–70. doi: 10.1161/CIRCRESAHA.117.310870 28404615 PMC5482785

[13] Wellen KE Fucho R Gregor MF Furuhashi M Morgan C Lindstad T. Coordinated Regulation of Nutrient and Inflammatory Responses by STAMP2 Is Essential for Metabolic Homeostasis. Cell (2007) 129:537–48. doi: 10.1016/J.CELL.2007.02.049 PMC2408881 17482547

[14] ten Freyhaus H Calay ES Yalcin A Vallerie SN Yang L Calay ZZ. Stamp2 Controls Macrophage Inflammation Through Nicotinamide Adenine Dinucleotide Phosphate Homeostasis and Protects Against Atherosclerosis. Cell Metab (2012) 16:81–9. doi: 10.1016/j.cmet.2012.05.009 PMC4163924 22704678

[15] Batool M Berghausen EM Zierden M Vantler M Schermuly RT Baldus S. The Six-Transmembrane Protein Stamp2 Ameliorates Pulmonary Vascular Remodeling and Pulmonary Hypertension in Mice. Basic Res Cardiol (2020) 115:68. doi: 10.1007/s00395-020-00826-8 33188479 PMC7666299

[16] Ohgami RS Campagna DR McDonald A Fleming MD. The Steap Proteins Are Metalloreductases. Blood (2006) 108:1388–94. doi: 10.1182/blood-2006-02-003681 PMC1785011 16609065

[17] Carrier L Schlossarek S Willis MS Eschenhagen T. The Ubiquitin-Proteasome System and Nonsense-Mediated mRNA Decay in Hypertrophic Cardiomyopathy. Cardiovasc Res (2010) 85:330–8. doi: 10.1093/cvr/cvp247 PMC4023315 19617224

[18] Kanno S Lerner DL Schuessler RB Betsuyaku T Yamada KA Saffitz JE. Echocardiographic Evaluation of Ventricular Remodeling in a Mouse Model of Myocardial Infarction. J Am Soc Echocardiogr (2002) 15:601–9. doi: 10.1067/mje.2002.117560 12050601

[19] English D Andersen BR. Single-Step Separation of Red Blood Cells, Granulocytes and Mononuclear Leukocytes on Discontinuous Density Gradients of Ficoll-Hypaque. J Immunol Methods (1974) 5:249–52. doi: 10.1016/0022-1759(74)90109-4 4427075

[20] Vettel C Lindner M Dewenter M Lorenz K Schanbacher C Riedel M. Phosphodiesterase 2 Protects Against Catecholamine-Induced Arrhythmia and Preserves Contractile Function After Myocardial Infarction. Circ Res (2017) 120:120–32. doi: 10.1161/CIRCRESAHA.116.310069 27799254

[21] Dick SA Macklin JA Nejat S Momen A Clemente-Casares X Althagafi MG. Self-Renewing Resident Cardiac Macrophages Limit Adverse Remodeling Following Myocardial Infarction. Nat Immunol (2019) 20:29–39. doi: 10.1038/s41590-018-0272-2 30538339 PMC6565365

[22] Travers JG Kamal FA Robbins J Yutzey KE Blaxall BC. Cardiac Fibrosis: The Fibroblast Awakens. Circ Res (2016) 118:1021–40. doi: 10.1161/CIRCRESAHA.115.306565 PMC4800485 26987915

[23] van den Borne SWM Diez J Blankesteijn WM Verjans J Hofstra L Narula J. Myocardial Remodeling After Infarction: The Role of Myofibroblasts. Nat Rev Cardiol (2010) 7:30–7. doi: 10.1038/nrcardio.2009.199 19949426

[24] Turner NA Blythe NM. Cardiac Fibroblast P38 MAPK: A Critical Regulator of Myocardial Remodeling. J Cardiovasc Dev Dis (2019) 6(3):27. doi: 10.3390/jcdd6030027 PMC6787752 31394846

[25] Prabhu SD Frangogiannis NG. The Biological Basis for Cardiac Repair After Myocardial Infarction: From Inflammation to Fibrosis. Circ Res (2016) 119:91–112. doi: 10.1161/CIRCRESAHA.116.303577 27340270 PMC4922528

[26] Ma Y Yabluchanskiy A Iyer RP Cannon PL Flynn ER Jung M. Temporal Neutrophil Polarization Following Myocardial Infarction. Cardiovasc Res (2016) 110:51–61. doi: 10.1093/cvr/cvw024 26825554 PMC4798046

[27] Liu T Zhang L Joo D Sun S-C. Nf-κb Signaling in Inflammation. Signal Transduct Target Ther (2017) 2:17023. doi: 10.1038/sigtrans.2017.23 29158945 PMC5661633

[28] Odobasic D Kitching AR Holdsworth SR. Neutrophil-Mediated Regulation of Innate and Adaptive Immunity: The Role of Myeloperoxidase. J Immunol Res (2016) 2016:2349817. doi: 10.1155/2016/2349817 26904693 PMC4745373

[29] Baxter GF. The Neutrophil as a Mediator of Myocardial Ischemia-Reperfusion Injury: Time to Move on. Basic Res Cardiol (2002) 97:268–75. doi: 10.1007/s00395-002-0366-7 12111036

[30] Vinten-Johansen J. Involvement of Neutrophils in the Pathogenesis of Lethal Myocardial Reperfusion Injury. Cardiovasc Res (2004) 61:481–97. doi: 10.1016/j.cardiores.2003.10.011 14962479

[31] Eltzschig HK Eckle T. Ischemia and Reperfusion–From Mechanism to Translation. Nat Med (2011) 17:1391–401. doi: 10.1038/nm.2507 PMC3886192 22064429

[32] Bouabdallaoui N Tardif J-C Waters DD Pinto FJ Maggioni AP Diaz R. Time-to-Treatment Initiation of Colchicine and Cardiovascular Outcomes After Myocardial Infarction in the Colchicine Cardiovascular Outcomes Trial (COLCOT). Eur Heart J (2020) 41(42):4092–9. doi: 10.1093/eurheartj/ehaa659 PMC7700755 32860034

[33] Tardif J-C Kouz S Waters DD Bertrand OF Diaz R Maggioni AP. Efficacy and Safety of Low-Dose Colchicine After Myocardial Infarction. N Engl J Med (2019) 381:2497–505. doi: 10.1056/NEJMoa1912388 31733140

[34] Ridker PM Everett BM Thuren T MacFadyen JG Chang WH Ballantyne C. Antiinflammatory Therapy With Canakinumab for Atherosclerotic Disease. N Engl J Med (2017) 377:1119–31. doi: 10.1056/NEJMoa1707914 28845751

[35] Wang F Han L Qin R Zhang Y Wang D Wang Z-H. Overexpressing STAMP2 Attenuates Adipose Tissue Angiogenesis and Insulin Resistance in Diabetic Apoe ^–/–^ /LDLR ^–/–^ Mouse. via PPARγ/CD36 pathway. J Cell Mol Med (2017) 21:3298–308. doi: 10.1111/jcmm.13233 PMC5706521 28631352

[36] Han L Tang M-X Ti Y Wang Z-H Wang J Ding W-Y. Overexpressing STAMP2 Improves Insulin Resistance in Diabetic Apoe–/–/LDLR–/– Mice *via* Macrophage Polarization Shift in Adipose Tissues. PloS One (2013) 8:e78903. doi: 10.1371/journal.pone.0078903 24236066 PMC3827284

[37] Jin Y Wang L Qu S Sheng X Kristian A Mælandsmo GM. STAMP 2 Increases Oxidative Stress and is Critical for Prostate Cancer. EMBO Mol Med (2015) 7:315–31. doi: 10.15252/emmm.201404181 PMC4364948 25680860

[38] Castro-Alcaraz S Miskolci V Kalasapudi B Davidson D Vancurova I. NF-Kappa B Regulation in Human Neutrophils by Nuclear I Kappa B Alpha: Correlation to Apoptosis. J Immunol (2002) 169:3947–53. doi: 10.4049/jimmunol.169.7.3947 12244195

[39] Lacy P. Mechanisms of Degranulation in Neutrophils. Allergy Asthma Clin Immunol (2006) 2:98–108. doi: 10.1186/1710-1492-2-3-98 20525154 PMC2876182

[40] Davies MJ. Myeloperoxidase-Derived Oxidation: Mechanisms of Biological Damage and Its Prevention. J Clin Biochem Nutr (2011) 48:8–19. doi: 10.3164/jcbn.11-006FR 21297906 PMC3022070

[41] O’Rourke SA Dunne A Monaghan MG. The Role of Macrophages in the Infarcted Myocardium: Orchestrators of ECM Remodeling. Front Cardiovasc Med (2019) 6:101. doi: 10.3389/fcvm.2019.00101 31417911 PMC6685361

[42] Oh YJ Kim HY Lee MH Suh SH Choi Y Nam T. Cilostazol Improves HFD-Induced Hepatic Steatosis by Upregulating Hepatic STAMP2 Expression Through AMPK. Mol Pharmacol (2018) 94:1401–11. doi: 10.1124/mol.118.113217 30366981

[43] Luo T Zeng X Yang W Zhang Y. Treatment With Metformin Prevents Myocardial Ischemia-Reperfusion Injury *via* STEAP4 Signaling Pathway. Anatol J Cardiol (2019) 21:261–71. doi: 10.14744/AnatolJCardiol.2019.11456 PMC6528516 31062756

