# Supplementary material for: Stamp2 Protects From Maladaptive Structural Remodeling and Systolic Dysfunction in Post-Ischemic Hearts by Attenuating Neutrophil Activation
Source: Front Immunol. 2021 Oct 6;12:701721. doi: 10.3389/fimmu.2021.701721 (PMC8527169; doi:10.3389/fimmu.2021.701721)
Supplement: Supplementary file 1 [file Presentation_1.pptx]

## Slide 1
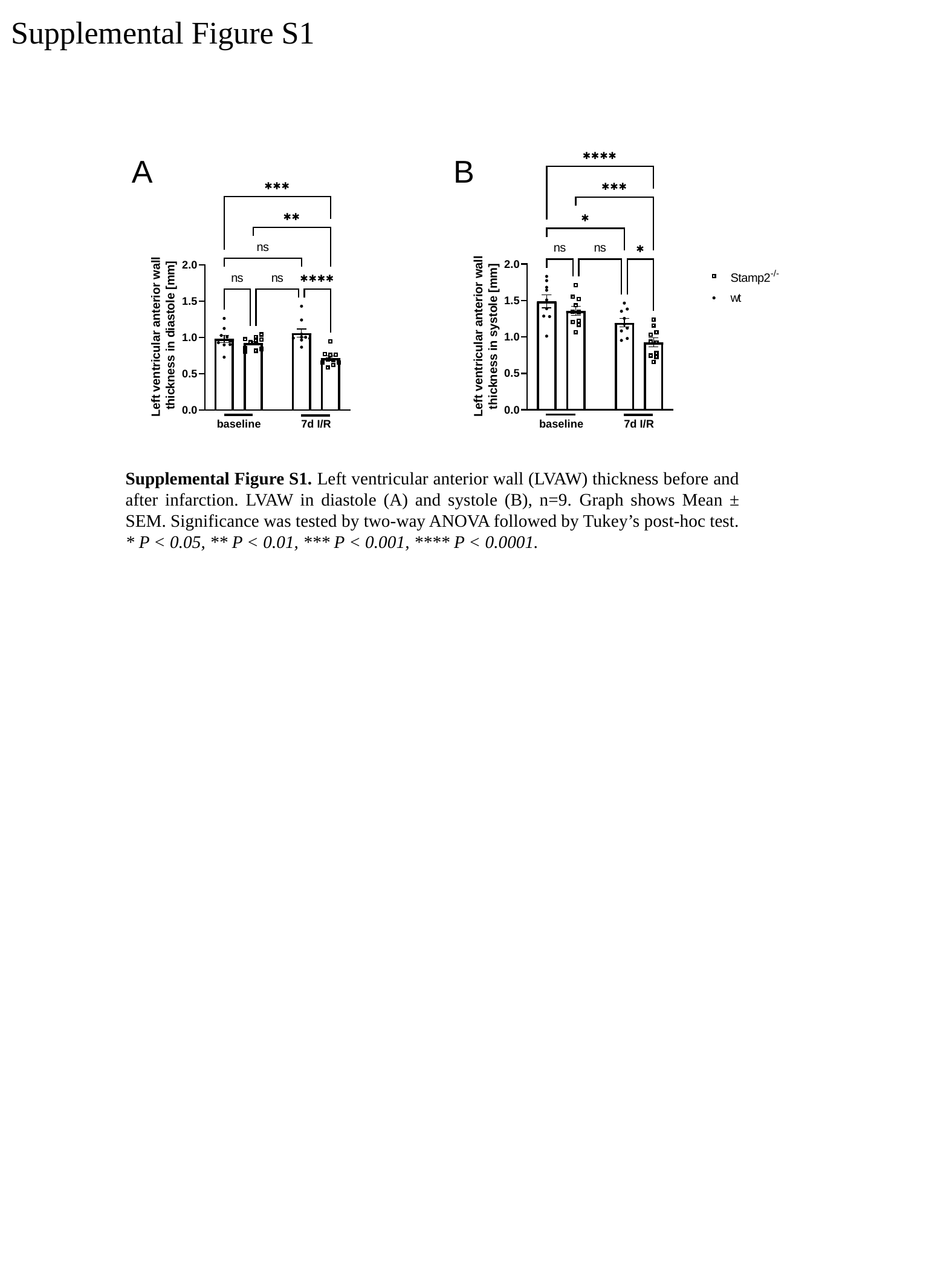

Supplemental Figure S1
A
B
Supplemental Figure S1. Left ventricular anterior wall (LVAW) thickness before and after infarction. LVAW in diastole (A) and systole (B), n=9. Graph shows Mean ± SEM. Significance was tested by two-way ANOVA followed by Tukey’s post-hoc test. * P < 0.05, ** P < 0.01, *** P < 0.001, **** P < 0.0001.

## Slide 2
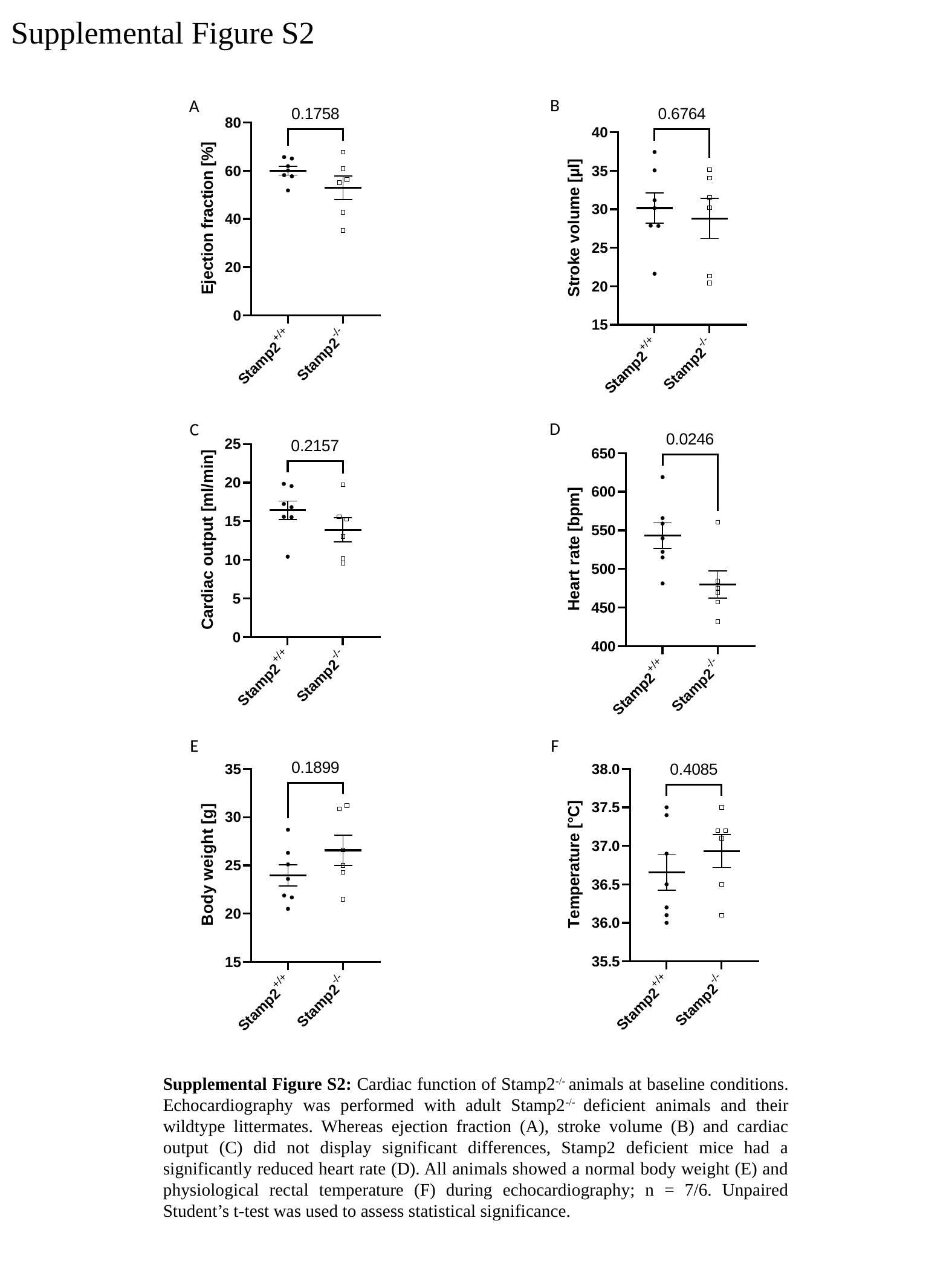

Supplemental Figure S2
B
A
D
C
E
F
Supplemental Figure S2: Cardiac function of Stamp2-/- animals at baseline conditions. Echocardiography was performed with adult Stamp2-/- deficient animals and their wildtype littermates. Whereas ejection fraction (A), stroke volume (B) and cardiac output (C) did not display significant differences, Stamp2 deficient mice had a significantly reduced heart rate (D). All animals showed a normal body weight (E) and physiological rectal temperature (F) during echocardiography; n = 7/6. Unpaired Student’s t-test was used to assess statistical significance.

## Slide 3
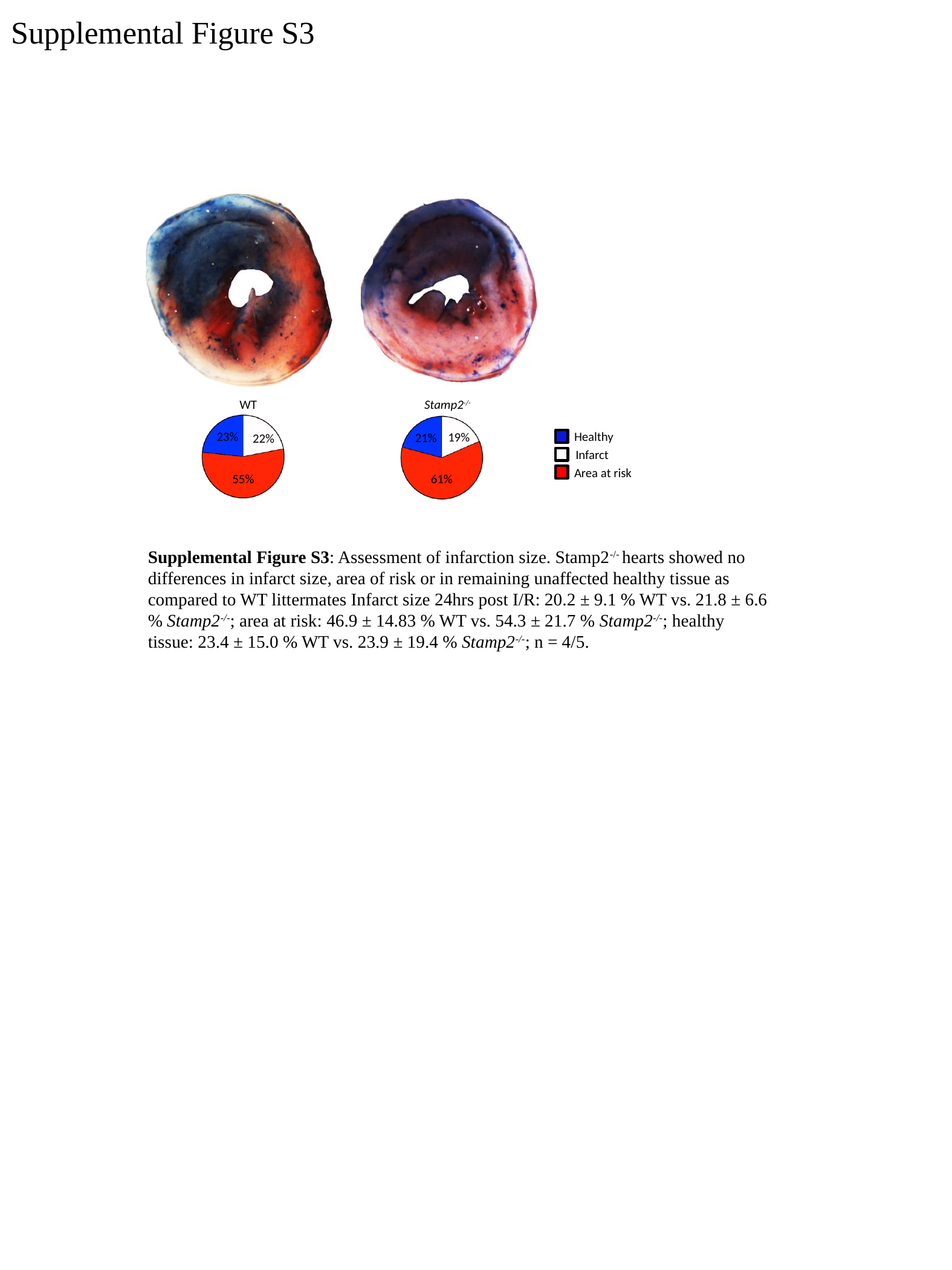

Supplemental Figure S3
WT
Stamp2-/-
23%
Healthy
19%
21%
22%
Infarct
Area at risk
55%
61%
Supplemental Figure S3: Assessment of infarction size. Stamp2-/- hearts showed no differences in infarct size, area of risk or in remaining unaffected healthy tissue as compared to WT littermates Infarct size 24hrs post I/R: 20.2 ± 9.1 % WT vs. 21.8 ± 6.6 % Stamp2-/-; area at risk: 46.9 ± 14.83 % WT vs. 54.3 ± 21.7 % Stamp2-/-; healthy tissue: 23.4 ± 15.0 % WT vs. 23.9 ± 19.4 % Stamp2-/-; n = 4/5.

## Slide 4
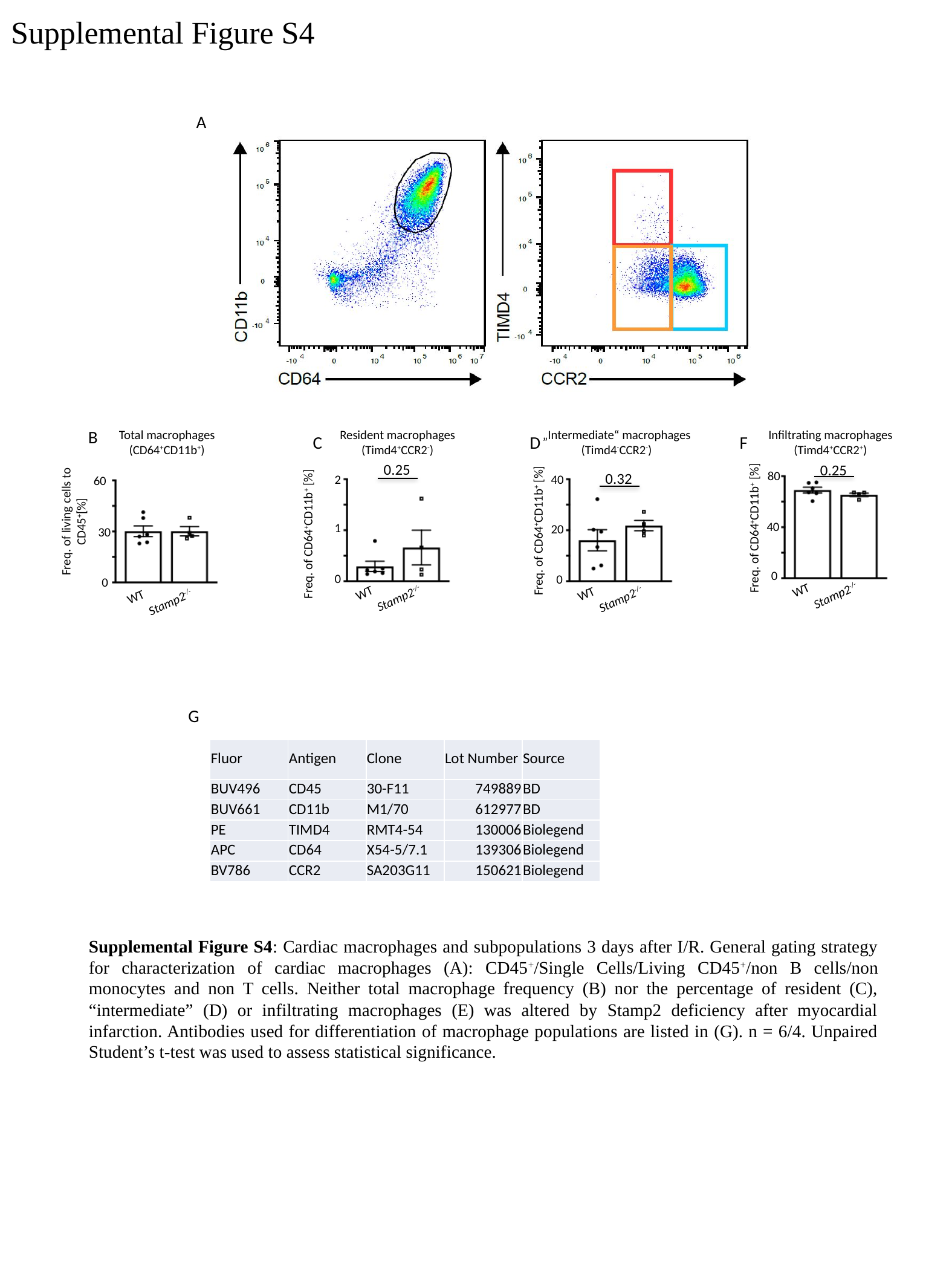

Supplemental Figure S4
A
B
Total macrophages
(CD64+CD11b+)
Resident macrophages
(Timd4+CCR2-)
„Intermediate“ macrophages
(Timd4-CCR2-)
Infiltrating macrophages
(Timd4+CCR2+)
C
D
F
0.25
0.25
80
0.32
2
40
60
Freq. of living cells to CD45+[%]
40
1
Freq. of CD64+CD11b+ [%]
20
Freq. of CD64+CD11b+ [%]
30
Freq. of CD64+CD11b+ [%]
0
0
0
0
WT
WT
WT
Stamp2-/-
WT
Stamp2-/-
Stamp2-/-
Stamp2-/-
G
| Fluor | Antigen | Clone | Lot Number | Source |
| --- | --- | --- | --- | --- |
| BUV496 | CD45 | 30-F11 | 749889 | BD |
| BUV661 | CD11b | M1/70 | 612977 | BD |
| PE | TIMD4 | RMT4-54 | 130006 | Biolegend |
| APC | CD64 | X54-5/7.1 | 139306 | Biolegend |
| BV786 | CCR2 | SA203G11 | 150621 | Biolegend |
Supplemental Figure S4: Cardiac macrophages and subpopulations 3 days after I/R. General gating strategy for characterization of cardiac macrophages (A): CD45+/Single Cells/Living CD45+/non B cells/non monocytes and non T cells. Neither total macrophage frequency (B) nor the percentage of resident (C), “intermediate” (D) or infiltrating macrophages (E) was altered by Stamp2 deficiency after myocardial infarction. Antibodies used for differentiation of macrophage populations are listed in (G). n = 6/4. Unpaired Student’s t-test was used to assess statistical significance.

## Slide 5
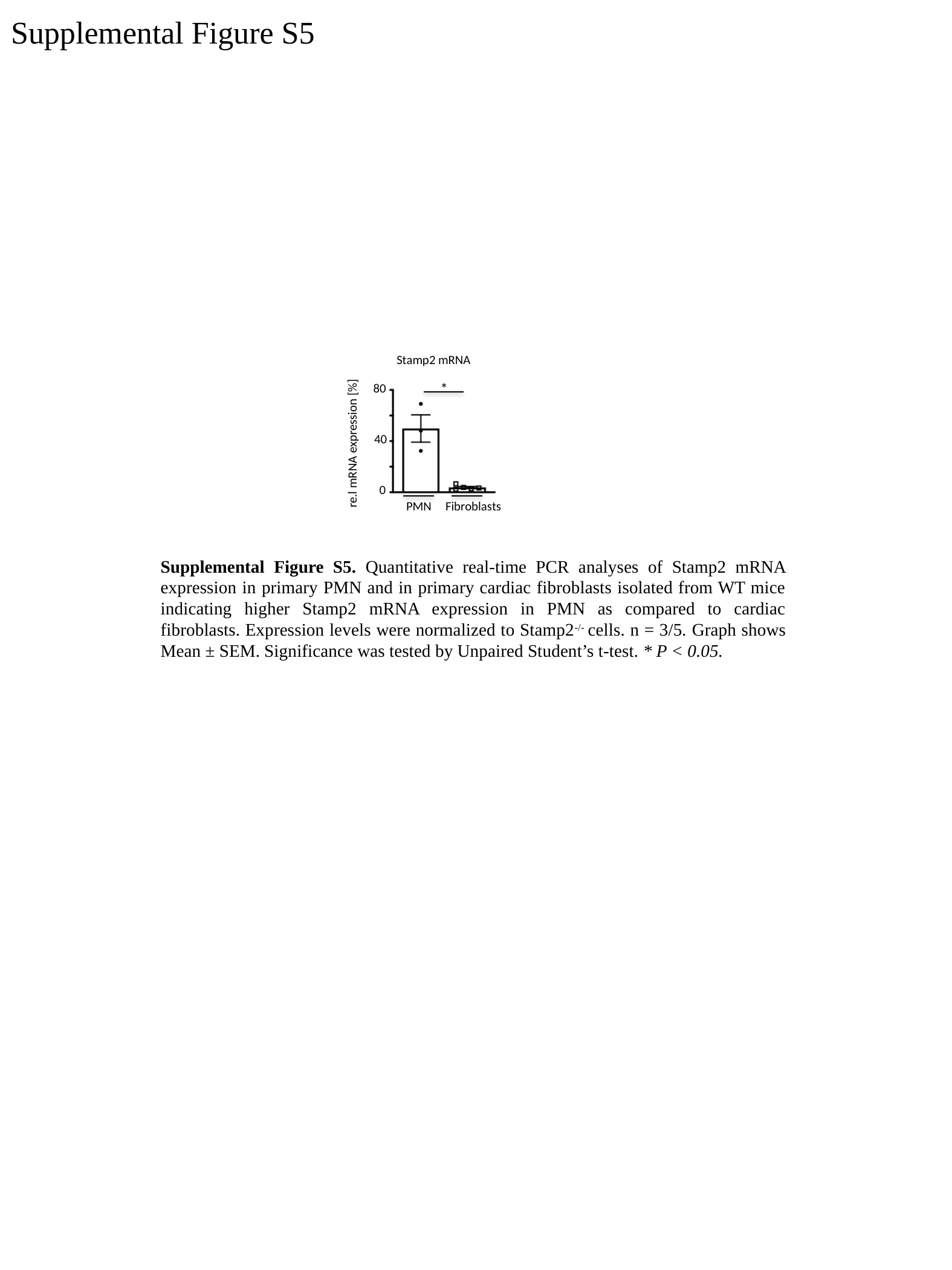

Supplemental Figure S5
Stamp2 mRNA
*
80
40
re.l mRNA expression [%]
0
PMN
Fibroblasts
Supplemental Figure S5. Quantitative real-time PCR analyses of Stamp2 mRNA expression in primary PMN and in primary cardiac fibroblasts isolated from WT mice indicating higher Stamp2 mRNA expression in PMN as compared to cardiac fibroblasts. Expression levels were normalized to Stamp2-/- cells. n = 3/5. Graph shows Mean ± SEM. Significance was tested by Unpaired Student’s t-test. * P < 0.05.

## Slide 6
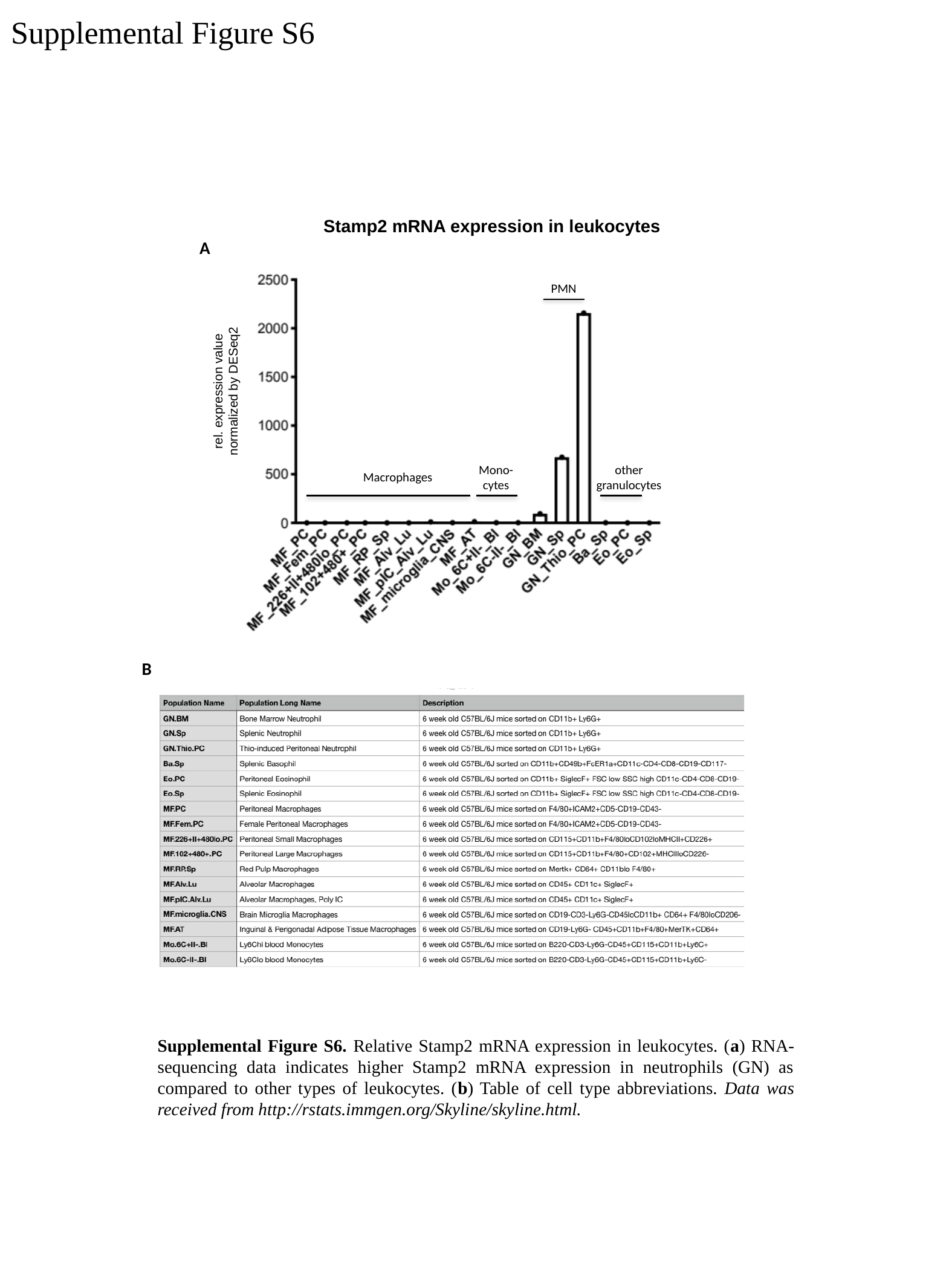

Supplemental Figure S6
Stamp2 mRNA expression in leukocytes
A
PMN
rel. expression value normalized by DESeq2
Mono-
cytes
other granulocytes
Macrophages
B
Supplemental Figure S6. Relative Stamp2 mRNA expression in leukocytes. (a) RNA-sequencing data indicates higher Stamp2 mRNA expression in neutrophils (GN) as compared to other types of leukocytes. (b) Table of cell type abbreviations. Data was received from http://rstats.immgen.org/Skyline/skyline.html.

## Slide 7
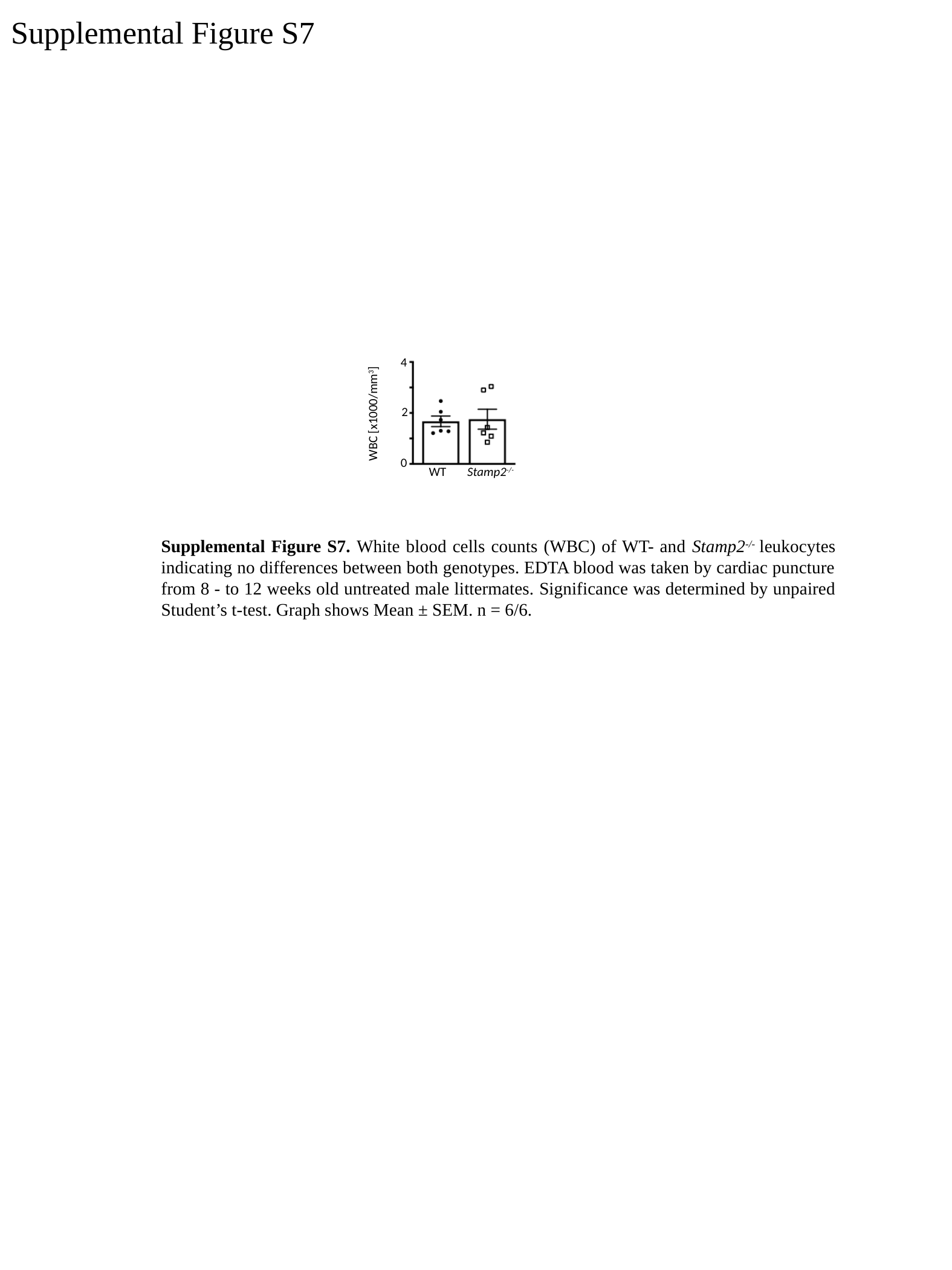

Supplemental Figure S7
4
2
WBC [x1000/mm3]
0
WT
Stamp2-/-
Supplemental Figure S7. White blood cells counts (WBC) of WT- and Stamp2-/- leukocytes indicating no differences between both genotypes. EDTA blood was taken by cardiac puncture from 8 - to 12 weeks old untreated male littermates. Significance was determined by unpaired Student’s t-test. Graph shows Mean ± SEM. n = 6/6.

## Slide 8
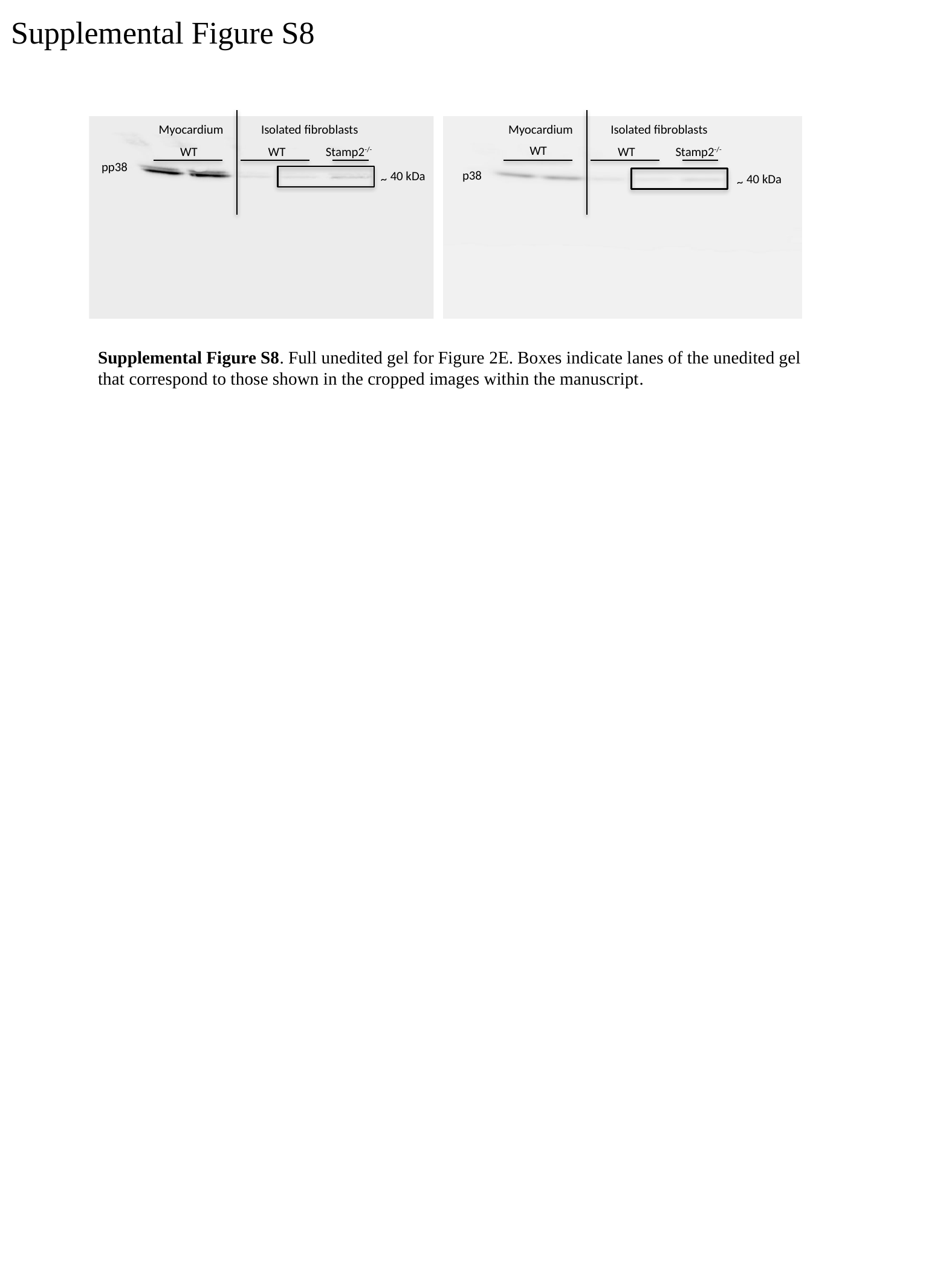

Supplemental Figure S8
Myocardium
Isolated fibroblasts
Myocardium
Isolated fibroblasts
WT
WT
WT
Stamp2-/-
WT
Stamp2-/-
pp38
p38
40 kDa
~
40 kDa
~
Supplemental Figure S8. Full unedited gel for Figure 2E. Boxes indicate lanes of the unedited gel that correspond to those shown in the cropped images within the manuscript.

## Slide 9
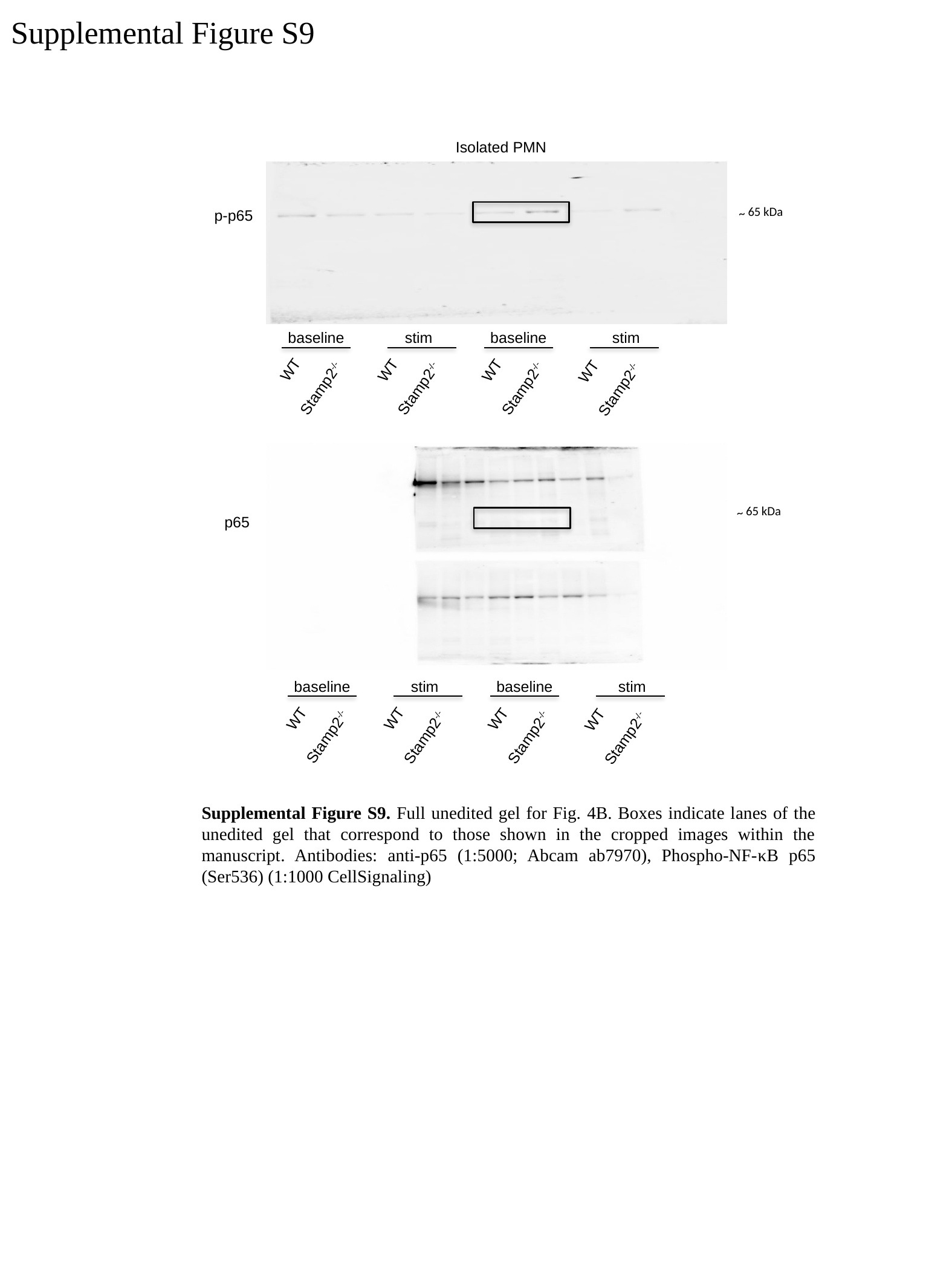

Supplemental Figure S9
Isolated PMN
65 kDa
p-p65
~
baseline
stim
baseline
stim
WT
WT
WT
WT
Stamp2-/-
Stamp2-/-
Stamp2-/-
Stamp2-/-
65 kDa
~
p65
baseline
stim
baseline
stim
WT
WT
WT
WT
Stamp2-/-
Stamp2-/-
Stamp2-/-
Stamp2-/-
Supplemental Figure S9. Full unedited gel for Fig. 4B. Boxes indicate lanes of the unedited gel that correspond to those shown in the cropped images within the manuscript. Antibodies: anti-p65 (1:5000; Abcam ab7970), Phospho-NF-κB p65 (Ser536) (1:1000 CellSignaling)
